# TRPC6 inactivation does not protect against diabetic kidney disease in streptozotocin (STZ)‐treated Sprague‐Dawley rats

**DOI:** 10.1096/fba.2019-00077

**Published:** 2019-12-02

**Authors:** Naghmeh Hassanzadeh Khayyat, Eun Young Kim, Stuart E. Dryer

**Affiliations:** ^1^ Department of Biology and Biochemistry University of Houston Houston TX USA; ^2^ Department of Biomedical Sciences University of Houston College of Medicine Houston TX USA

**Keywords:** diabetic nephropathy, ion channel, nephrin, podocyte

## Abstract

Canonical transient receptor potential‐6 (TRPC6) channels have been implicated in the progression of several forms of kidney disease (1). While there is strong evidence that glomerular TRPC6 channels are dysregulated in diabetic nephropathy (DN), there is no consensus as to whether deletion or inactivation of TRPC6 is protective in animal models of DN. A previous study in Dahl salt‐sensitive rats suggests that TRPC6 knockout has a modest protective effect in streptozotocin (STZ)‐induced DN (2). In the present study, we examined whether inactivation of TRPC6 channels by CRISPR/Cas9 editing (*Trpc6* ^del/del^ rats) affects progression of STZ‐induced DN in Sprague‐Dawley rats. Wild‐type littermates (*Trpc6* ^wt/wt^ rats) were used as controls. We observed that a single injection of STZ resulted in severe hyperglycemia that was sustained over a 10‐week period, accompanied by a marked reduction in circulating C‐peptide, dyslipidemia, and failure to gain weight compared to vehicle‐treated animals. Those effects were equally severe in *Trpc6* ^wt/wt^ and *Trpc6* ^del/del^ rats. STZ treatment resulted in increased urine albumin excretion at 4, 8, and 10 weeks after injection, and this effect was equally severe in *Trpc6* ^wt/wt^ and *Trpc6* ^del/del^ rats. TRPC6 inactivation had no effect on blood urea nitrogen (BUN), plasma creatinine concentration, urine nephrin excretion, or kidney weight:body weight ratio measured 10 weeks after STZ injection. STZ treatment evoked modest and equivalent mesangial expansion in *Trpc6* ^wt/wt^ and *Trpc6* ^del/del^ rats. In summary, we observed no protective effect of TRPC6 inactivation on STZ‐induced DN in rats on the Sprague‐Dawley background.

**Abbreviations**
BUN: blood urea nitrogen
Dahl SS: dahl salt-sensitive
DN: diabetic nephropathy
ESRD: end-stage renal disease
FSGS: focal and segmental glomerulosclerosis
GBM: glomerular basement membrane
GFR: glomerjlar filtration rate
NTS: nephrotoxic serum
PAN: puromycin aminonucleoside
PAS: periodic acid-Schiff’s
SMA: α-smooth muscle actin
STZ: streptozotocin
TRPC6: canonical transient receptor potential-6 channels

## 1 INTRODUCTION

Diabetic nephropathy (DN)(1, 2) is a major cause of end‐stage renal disease (ESRD).^3^, ^4^ Current therapies for DN entail intensive glycemic control and inhibition of renin‐angiotensin systems,^3^, ^5^ but in many cases, these therapies fail to prevent progression to ESRD.^6^ Consequently, it is important to identify new and plausible therapeutic targets for DN. The mechanisms driving progression of DN are complex, and treatment outcomes appear to be affected by genetic background.^7^ This is also reflected in animal models where the severity of DN is highly strain‐dependent.^8^, ^9^, ^10^, ^11^

Podocytes are among the earliest cell types affected in DN,^12^ and the proteinuria associated with DN is correlated with foot process effacement and subsequent detachment of podocytes from the glomerular basement membrane (GBM).^13^ Podocytes are highly differentiated polarized cells that do not readily regenerate, and therefore, podocyte loss affects the integrity of the glomerular filter and is thought to contribute to progression of glomerulosclerosis and renal dysfunction in a wide range of renal diseases.^14^, ^15^ While podocytes appear to be especially sensitive, diabetes induces functional changes in mesangial cells, glomerular endothelial cells, tubular cells, and vascular smooth muscle, and within the renal interstitium.^16^, ^17^

Canonical transient receptor potential‐6 channels are non‐selective Ca^2+^‐permeable cation channels expressed in many different cell types. We have recently reviewed the current understanding of the role of TRPC6 and related cationic channels in the pathophysiology of kidney disease, and their status as potential therapeutic targets.^1^ Podocyte TRPC6 channels are expressed at the slit diaphragm domains of foot processes^18^, ^19^ and along major processes and in the cell body.^1^, ^20^ They are also present in mesangial cells.^1^, ^21^ Gain‐of‐function mutations in the *Trpc6* gene are associated with familial forms of focal segmental glomerulosclerosis (FSGS).^19^, ^22^ TRPC6 dysregulation is also linked to progression of acquired forms of proteinuric kidney diseases.^1^, ^23^, ^24^, ^25^, ^26^ We have recently reported that podocyte TRPC6 channels are dysregulated in the chronic puromycin aminonucleoside (PAN) nephrosis model of acquired FSGS in Sprague‐Dawley rats,^1^, ^25^ as well as in response to circulating factors implicated in primary and recurrent FSGS.^24^, ^25^ Moreover, we have observed that TRPC6 inactivation exerts a marked renoprotective effect in chronic PAN nephrosis^25^ and, to a lesser extent, in the nephrotoxic serum (NTS) model of autoimmune glomerulonephritis.^27^

It has been widely reported that glomerular TRPC6 channels are substantially more abundant in type 1 and type 2 diabetes and in podocytes cultured in the presence of elevated external glucose.^28^, ^29^, ^30^, ^31^, ^32^, ^33^ This is due at least in part to oxidative stress that can be driven by hyperglycemia, and by the surrounding pro‐inflammatory milieu.^1^ In addition, a protective effect of *Trpc6* knockout has been reported in animal models of type 1 diabetes, although the outcomes varied substantially depending on which animal model was used. For example, a protective effect of *Trpc6* knockout was observed in the Akita mouse model of type 1 diabetes at 12 and 16 weeks of age. However, the protective effects declined after that, and by 20 weeks of age, the *Trpc6* knockout mice actually had more severe mesangial expansion than wild‐type controls.^34^ The gradual decline in protection conferred by *Trpc6* knockout was attributed to several factors, including progressive insulin resistance and increased renal expression of pro‐inflammatory signaling systems that occurred as *Trpc6* knockout animals became older.^34^ In a different study, a modest renoprotective effect was reported in the streptozotocin (STZ) model of type 1 diabetes in Dahl salt‐sensitive rats maintained on a normal diet (0.4% NaCl) in which *Trpc6* was deleted using CRISPR/Cas9 gene editing.^2^ In those experiments, *Trpc6* knockout rats exhibited a reduction in urine nephrin excretion, which suggests attenuation of diabetes‐induced podocyte detachment compared to wild‐type controls. These authors also reported a reduction in foot process effacement (although that effect was not quantified). On the other hand, they did not observe any reductions in albumin excretion or any change in light microscopic histology in diabetic *Trpc6* knockout rats.^2^

In the present study, we have investigated whether TRPC6 channels play a role in the progression of DN in STZ‐treated Sprague‐Dawley rats, a strain that has been widely used in studies on renal physiology and pathophysiology. In these experiments, we used Sprague‐Dawley rats in which TRPC6 channels were inactivated by a global constitutive deletion in exon 2 of the *Trpc6* gene generated by CRISPR/Cas9, which we have described previously.^25^ Rats homozygous for this deletion, hereafter referred to as *Trpc6* ^del/del^, exhibited marked protection from chronic PAN nephrosis compared to wild‐type littermate controls (*Trpc6* ^wt/wt^),^25^ and we hypothesized that these animals would also be protected from STZ‐induced nephropathy. However, in marked contrast to the predictions of that hypothesis, we were not able to discern either a protective or exacerbating effect of TRPC6 inactivation on renal function or on the diabetic phenotype at any time point that we examined following the initial STZ injection.

## 2 MATERIALS AND METHODS

### 2.1 Animals

All animal procedures were conducted according to protocols approved by the University of Houston Institutional Animal Care and Use Committee (IACUC) following National Institutes of Health and Animal Research: Reporting of In Vivo Experiments guidelines. These studies used male *Trpc6* ^del/del^ rats at 8‐9 weeks of age and male *Trpc6* ^wt/wt^ littermates, which we have described previously.^25^ Briefly, CRISPR/Cas9 methods were used to produce a 239‐bp deletion within exon 2 of the *Trpc6* gene, which encodes an essential portion of the ankyrin repeat domain of the *Trpc6* gene. As a consequence of this deletion, all of exon 2 was spliced out of the *Trpc6* transcripts, resulting in non‐functional channels.^25^

### 2.2 Streptozotocin (STZ)‐induced diabetes

Rats were weighed and placed in metabolic cages for collection of 12‐hr urine samples, which were used to obtain baseline measures of renal function. Two days later, rats were administered a single i.p. injection of STZ (65 mg/kg in 0.1 mol/L Na‐citrate buffer, pH 4.5) or 0.1 mol/L Na‐citrate vehicle (pH 4.5). Animals did not receive any exogenous insulin after the STZ injection. Blood was collected via the lateral tail vein five days after injections, and STZ‐treated rats with hyperglycemia >450 mg/dL, and all of the vehicle‐treated animals, were monitored over the next 10 weeks. Additional blood samples were collected at four and ten weeks following STZ or vehicle injections to assess the progression of diabetes and to monitor renal function by measurements of blood urea nitrogen (BUN) and plasma creatinine. Urine samples were also collected at various times following the injections, and urine albumin and nephrin levels were quantified by ELISAs (Ethos Biosciences Inc), whereas creatinine was quantified using a colorimetric assay based on the Jaffe reaction (Ethos Biosciences). At the end of the 10‐week protocol, animals were euthanized by CO_2_ inhalation followed by cervical dislocation, kidneys were excised and weighed, and the left renal cortex was used for various immunoblot analyses. The right kidneys were used for histological analysis as described previously^25^ and further below.

### 2.3 Immunoblot analysis and enzyme‐linked immunosorbent assays

The cortex of the left kidney was diced into small pieces, lysed in 1 mL of M‐PER™ mammalian protein extraction buffer (Thermo Fisher Scientific) containing Protease Inhibitor Cocktail (Sigma‐Aldrich), and homogenized by sonication on ice. Homogenates were clarified by centrifugation at 15 115 *g* for 15 minutes at 4°C, and immunoblot analyses of the supernatants were carried out using standard methods as described previously.^24^ Antibodies used for immunoblot were mouse monoclonal anti‐TRPC6 (sc‐515837, Santa Cruz Biotechnology, used at 1:200); mouse monoclonal anti‐TRPC5 (NeuroMAB, N67/15, used at 1:200); rabbit polyclonal anti‐podocin (PA5‐79757, Thermo Fisher Scientific, used at 1:500); mouse monoclonal anti‐β‐actin (AC004, ABclonal, used at 1:5000); and mouse monoclonal anti‐α‐smooth muscle actin (α‐SMA) (clone 1A4, A2547, Sigma‐Aldrich, used at 1:500). Immunoblot experiments were performed in triplicate and quantified by densitometry using ImageJ^TM^ software. Plasma C‐peptide was measured by ELISA (#80‐COTRT‐E01; ALPCO, Salem, NH), and plasma creatinine was measured using an enzymatic assay (Crystal Chem, Elk Grove Village, IL). Urine creatinine was measured using a colorimetric assay based on the Jaffe reaction (Ethos Biosciences). BUN, glucose, triglycerides, and total cholesterol were measured using standard clinical methods at the Baylor College of Medicine Metabolic and Phenotyping Core Facility.

### 2.4 Histopathology

The right kidney was fixed in 10% buffered formalin, embedded in paraffin, and 4‐μmol/L sections were cut and stained with periodic acid‐Schiff (PAS).^25^ To assess the glomerular injury in PAS‐stained slides, 50 glomeruli per animal for all animals in all groups (N = 7‐9 rats per group) were picked at random and mesangial matrix expansion was scored by a blind observer on a scale of 0‐3 as described previously.^35^ Briefly, 0 = normal glomeruli; 1 = glomeruli with 25%‐50% of their volume occupied by mesangial matrix; 2 = glomeruli with 51%‐75% of their volume occupied by mesangial matrix; and 3 = glomeruli with over 76% of their volume occupied by mesangial matrix. For each animal, at least 50 glomeruli were evaluated and used to acquire a mean value for that animal. Statistical analyses were then carried out using the animal means for all animals of a given genotype and treatment group.

### 2.5 Statistical analyses

All statistical analyses were carried out using the online computational tools at (http://www.vassarstats.net) with *α = *.05. Data from urine and plasma measurements were analyzed by two‐way ANOVA. The two independent variables were genotype (*Trpc6* ^wt/wt^ vs *Trpc6* ^del/del^) and drug treatment (STZ vs vehicle). To assess whether the magnitude of any of the STZ effects was affected by TRPC6 inactivation, the key statistical parameters are the *F* and *P* values for the interaction between drug effects and genotype. Immunoblot data in bar graphs are presented as mean ± SEM of triplicate independent measurements, with the ordinate normalized to the lowest value observed in a control group.

## 3 RESULTS

**Figure 1:**
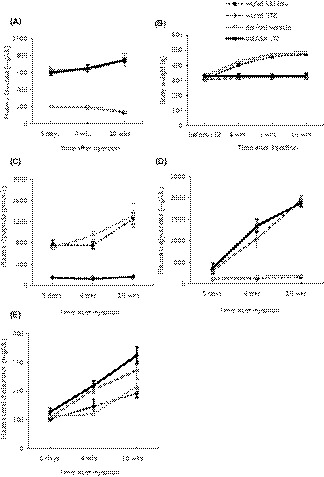
Characteristics of streptozotocin (STZ)‐induced diabetes in Sprague‐Dawley rats. Rats were given a single injection of STZ or vehicle and then followed over time. A, STZ‐injected rats exhibited hyperglycemia throughout the course of the experiment, and this was equivalent in *Trpc6* ^wt/wt^ and *Trpc6* ^del/del^ rats. Vehicle‐injected rats of both genotypes exhibited normal and stable blood glucose concentration. B, Vehicle‐injected rats continued to gain weight over the next 10 wk, but STZ‐injected rats did not gain weight. There was no discernible difference between *Trpc6* ^wt/wt^ and *Trpc6* ^del/del^ rats. C, Plasma C‐peptide was very low in STZ‐injected rats but not in vehicle‐injected rats. For unknown reasons, plasma C‐peptide levels increased between 4 and 8 wk of age, but this happened in both *Trpc6* ^wt/wt^ and *Trpc6* ^del/del^ rats. D, STZ treatment resulted in increased plasma triglycerides in *Trpc6* ^wt/wt^ and *Trpc6* ^del/del^ rats. E, STZ treatment also increased plasma total cholesterol in *Trpc6* ^wt/wt^ and *Trpc6* ^del/del^ rats. In this and all subsequent figures, data are presented as mean ± SEM

Experiments in this study were carried out on *Trpc6* ^del/del^ rats that were created on a Sprague‐Dawley background, which have been described previously.^25^ Briefly, a 239‐bp deletion was introduced into exon 2 of the *Trpc6* gene, resulting in deletion of the entire exon after transcription. The resulting truncated TRPC6 channels (which can be detected in glomeruli at very low levels) are non‐functional.^25^ Wild‐type (*Trpc6* ^wt/wt^) littermates were used as controls. Experiments were carried out on four groups of animals: vehicle‐treated *Trpc6* ^wt/wt^ rats; STZ‐treated *Trpc6* ^wt/wt^ rats; vehicle‐treated *Trpc6* ^del/del^ rats; and STZ‐treated *Trpc6* ^del/del^ rats. Diabetes was induced by a single intraperitoneal injection of STZ in both *Trpc6* ^wt/wt^ and *Trpc6* ^del/del^ rats. Plasma glucose levels were monitored at 5 days, 4 weeks, and 10 weeks following STZ injections. STZ‐treated rats exhibited severe hyperglycemia by 5 days after STZ injection, and this was apparent throughout the period of the experiment, whereas vehicle‐treated control animals exhibited stable blood glucose (Figure 1A). STZ‐treated rats of both genotypes failed to gain weight following STZ injection, whereas vehicle‐treated controls exhibited a >50% increase in body weight over the 10 weeks following injection (Figure 1B). Consistent with this, C‐peptide levels were reduced in all STZ‐treated rats (Figure 1C and Figure S1). All of the vehicle‐treated rats, regardless of their genotype, exhibited a noticeable increase in C‐peptide between 4 and 10 weeks after initial injections, and we do not know why this occurred. STZ‐treated rats exhibited a significant increase in plasma triglycerides and total cholesterol concentrations at 4 and 10 weeks after STZ administration (Figure 1D,E and Figure S1), and again, this did not depend on genotype. The absence of functional TRPC6 channels had no effect on any of these measurements, and two‐way ANOVA based on measurements made 10 weeks after injections did not discern any interaction between the effects of genotype and responses to STZ (Figure S1).

**Figure 2:**
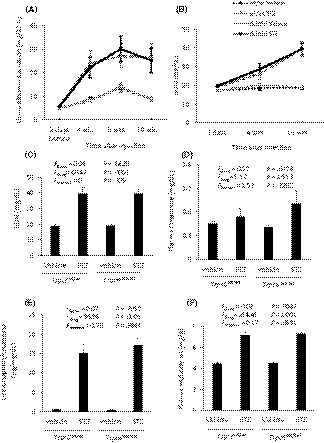
Changes in renal function during streptozotocin (STZ)‐induced diabetes in Sprague‐Dawley rats. A, Urine albumin excretion increased at 4 to 10 wk following STZ injection. This effect was equally severe in *Trpc6* ^wt/wt^ and *Trpc6* ^del/del^ rats. B, STZ treatment resulted in gradual increase in blood urea nitrogen (BUN) in STZ‐injected *Trpc6* ^wt/wt^ and *Trpc6* ^del/del^ rats, but there was no change in vehicle‐treated controls. C, Analysis of BUN in *Trpc6* ^wt/wt^ and *Trpc6* ^del/del^ rats at 10 wk following STZ or vehicle injection revealed a robust effect of the drug treatment but no effect of genotype. Two‐way ANOVA revealed no interaction between effects of STZ and genotype, indicating no protective effect of TRPC6 inactivation. D, A similar pattern was discerned from measurements of plasma creatinine. E, STZ treatment evoked an increase in urine nephrin excretion measured at 10 wk following injections by ELISA and normalized to urine creatinine, but two‐way ANOVA revealed no protective effect of TRPC6 inactivation. F, STZ injection resulted in increased kidney weight: body weight ratio at the end of the experiment, but two‐way ANOVA revealed no protective effect of TRPC6 inactivation

Streptozotocin‐treated rats exhibited changes in renal function. A baseline 12‐hr urine sample was collected from all animals two days prior to STZ or vehicle injections. STZ‐treated animals had marked increases in 12‐hr urine albumin excretion at 4, 8, and 10 weeks following injection compared to vehicle‐treated controls, and this pattern appeared virtually identical in *Trpc6* ^wt/wt^ and *Trpc6* ^del/del^ rats (Figure 2A). A similar pattern was observed in measurements of BUN (Figure 2B,C). Plasma creatinine (Figure 2D), urine nephrin excretion (Figure 2E), and kidney weight: body weight ratios (Figure 2F) measured at 10 weeks following injections were also increased in STZ‐treated animals. However, two‐way ANOVA revealed no significant interaction between effects of genotype and STZ on any of these parameters. In summary, STZ treatment resulted in a severe diabetic phenotype accompanied by declines in renal function, but there was no evidence of a protective effect of TRPC6 inactivation at any point during the 10‐week course of the experiment.

**Figure 3:**
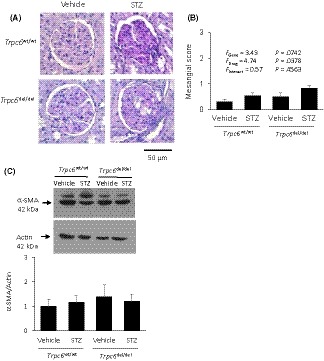
Canonical transient receptor potential‐6 (TRPC6) inactivation did not affect course of renal pathology. A, Typical examples of PAS‐stained glomeruli from streptozotocin (STZ)‐ or vehicle‐treated *Trpc6* ^wt/wt^ and *Trpc6* ^del/del^ rats as indicated. B, Semi‐quantitative analysis of mesangial expansion in glomeruli carried out by an observer blind to treatment group or genotype. Two‐way ANOVA indicates increase in mesangial expansion in STZ‐treated animals with no interaction between drug treatment and genotype. There is a marked trend toward increased mesangial expansion in *Trpc6* ^del/del^ rats. C, Immunoblot analysis of α‐SMA revealed no effect of either STZ treatment or genotype, suggesting that fibrosis in these animals was minimal

**Figure 4:**
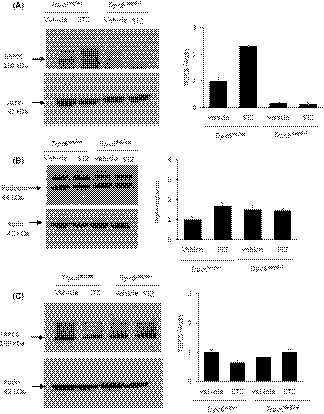
Abundance of Canonical transient receptor potential‐6 (TRPC6) and podocin in renal cortex. A, Immunoblot analysis showing increase in TRPC6 content at 10 wk after streptozotocin (STZ) injection in *Trpc6* ^wt/wt^ rats, and extremely low abundance of truncated TRPC6 in *Trpc6* ^del/del^ rats as described previously.^25^ A typical immunoblot showing results from representative animals is shown to the left, whereas densitometric analysis from the groups of animals is shown to the right. Note that those remaining TRPC6 proteins are not functional.^25^ B, There was no consistent effect of drug treatment or genotype on podocin abundance of renal cortex. A typical immunoblot showing results from representative animals is shown to the left, whereas densitometric analysis from the groups of animals is shown to the right. C, There was no consistent effect of drug treatment or genotype on TRPC5 abundance of renal cortex. A typical immunoblot showing results from representative animals is shown to the left, whereas densitometric analysis from the groups of animals is shown to the right

Animals were euthanized after completion of the 10‐week protocol, and mesangial expansion was quantified by an observer blind to the treatment group from PAS‐stained sections. The effects of STZ on renal histology were relatively minor compared to those that we have seen in other kidney disease models in these rat strains.^25^, ^27^ STZ treatment resulted in mesangial matrix expansion and modest interstitial hypercellularity in all of the STZ‐treated animals (Figure 3A). Glomerulosclerosis was rare. There were occasional regions where tubular atrophy and hyalinization could be detected in STZ‐treated animals. Glomeruli were scored on a semi‐quantitative scale of 0‐3 based on the severity of mesangial matrix expansion.^35^ These analyses revealed a modest but statistically significant effect of STZ, consistent with previous reports.^4^, ^28^, ^36^, ^37^, ^38^ However, there was no evidence of a protective effect of TRPC6 inactivation on glomerular pathology, and indeed, there was a trend toward an increase in mesangial matrix expansion simply as a result of TRPC6 inactivation (Figure 3A,B). In these animals, fibrosis was minimal as assessed by immunoblot analysis of α‐smooth muscle actin (α‐SMA) content (Figure 3C). Consistent with previous reports, we observed that STZ treatment resulted in an increase in the overall abundance of TRPC6 protein in renal cortex in *Trpc6* ^wt/wt^ rats (Figure 4A). Note that low levels of non‐functional TRPC6 protein are detectable in *Trpc6* ^wt/wt^ rats due to expression of *Trpc6* transcripts lacking exon 2. Podocin levels were not affected by STZ treatment or by TRPC6 inactivation (Figure 4B). Given that STZ treatment resulted in increased urine nephrin excretion (Figure 2E), this suggests that the remaining glomerular podocytes were hypertrophic.^39^ It bears noting that TRPC5 channels have also been implicated in the pathogenesis of chronic kidney disease.^1^, ^40^, ^41^ Here, we note that there was no change in renal cortical TRPC5 abundance as a result of TRPC6 inactivation, STZ treatment, or both (Figure 4C).

## 4 DISCUSSION

It has previously been shown that glomerular TRPC6 channels are upregulated in animal models of diabetes^28^, ^31^ and following exposure of podocytes to high glucose in vitro.^29^ It is now well established that sustained upregulation of TRPC6 can drive the progression of glomerular disease. Thus, gain‐of‐function mutations of TRPC6 result in severe familial FSGS that most typically occurs with an adult onset in humans.^19^, ^22^ Mice selectively over‐expressing either wild‐type or mutant TRPC6 in podocytes similarly exhibit albuminuria, foot process effacement, and glomerulosclerosis.^42^ Conversely, inactivation of TRPC6 using CRISPR/Cas9 methods reduces all aspects of kidney disease in the chronic PAN nephrosis model of FSGS in Sprague‐Dawley rats,^25^ and reduces glomerular disease caused by sustained angiotensin II infusions lasting up to three weeks in mice.^43^, ^44^ TRPC6 inactivation also reduced glomerulosclerosis in anti‐GBM autoimmune glomerulonephritis in rats.^27^ The purpose of the present study was to examine whether TRPC6 inactivation produces a similar protective effect in a rat model of diabetes.

A possible role for TRPC6 in driving DN has been examined by other investigators, but a consensus has not yet emerged, probably because of differences in experimental models. For example, using a standard low‐dose STZ model in mice, it was observed that triple knockout of the known diacylglycerol‐responsive TRPC channels (TRPC3, TRPC6, and TRPC7) reduced glomerular hypertrophy, albuminuria, and podocyte loss in diabetic animals, and also attenuated pro‐inflammatory TGF‐β signaling in glomeruli.^29^ Hyperglycemia was comparable in STZ‐treated triple knockout and wild‐type animals. However, triple knockout animals exhibited marked reductions in body weight (by 15%‐18%), and this effect was additive with the reductions in weight gain that typically occur in STZ diabetes in mice. With this triple knockout model, it is not possible to infer that protective effects are due to TRPC6 deletion.

A more recent study examined the effects of a single global and constitutive *Trpc6* knockout in the Akita mouse model of type I diabetes.^34^ These workers observed reductions in albuminuria in Akita mice with *Trpc6* knockout at 12 and 16 weeks of age compared to Akita mice that expressed wild‐type TRPC6 channels. However, this protective effect was no longer detected by 20 weeks of age, at which point mesangial expansion was actually more severe in *Trpc6* knockout Akita mice. These authors reported enhanced p38 and cyclooxygenase 2 signaling along with gradual development of insulin resistance in *Trpc6* knockout mice, which overcame the initial protective effects observed when the animals were younger.^34^

A modest protective effect of global *Trpc6* knockout has also been reported in Dahl salt‐sensitive (Dahl SS) rats treated with a single injection of STZ.^2^ It is important to note that in that study, there were no improvements in urine albumin excretion, glomerular injury, glomerular fibrosis, tubular protein casts, or glomerular filtration rate (GFR) in *Trpc6* knockout Dahl SS rats. However, these workers observed a reduction in foot process effacement (although this was not quantified) along with a reduction in urine nephrin content in STZ‐treated *Trpc6* knockout animals.^2^ The later measurement can be used to infer podocyte detachment, and this pattern suggests a modest protective effect of the *Trpc6* knockout. The animals in that study were maintained on a 0.4% NaCl diet and were therefore likely to be normotensive, although blood pressures were not reported.

Our experiments were carried out in rats on a Sprague‐Dawley background, which has been more extensively used in previous studies of glomerular function. Our experiments were carried out at nearly the same age as those performed by Spires et al,^2^ as they followed their animals for 11 weeks following STZ, whereas we monitored animals for 10 weeks. Compared to the studies of Spires et al,^2^ hyperglycemia in our animals was somewhat more severe during the first 28 days following STZ injection but was similar by the end of the experiment (10 weeks after STZ injection). In this study, we did not see any protective effect of TRPC6 inactivation by any measure, notably including quantitative analyses of urine nephrin levels, which were significantly elevated in STZ‐treated *Trpc6* ^wt/wt^ and *Trpc6* ^del/del^ rats by 10 weeks after STZ. The main experimental difference between our studies and that of Spires et al^2^ is the genetic background of the rats used for study. In both animal models, the CRISPR/Cas9 procedures resulted in complete loss of TRPC6 function. In both cases, TRPC6 inactivation had no impact on urine albumin excretion or light‐level histology of renal cortex. The principle difference is in our conclusions regarding podocyte detachment based on analyses of urine nephrin levels. Here, we will note that we measured nephrin using a commercial ELISA that was recently optimized for use in rats, whereas Spires et al^2^ used immunoblot analyses of urine. We do not think that this can explain the different conclusions, although it is somewhat surprising that Spires et al observed significant changes in urine nephrin without corresponding changes in urine albumin excretion.^2^

Genetic background is a major issue in the context of DN, but it has been most extensively and quantitatively studied in mice.^8^, ^9^, ^10^, ^11^ It is now clear that there is large variability in susceptibility of widely used inbred mouse strains to albuminuria and glomerulosclerosis in DN. Moreover, susceptibility loci for DN have been identified in humans.^45^ It is possible that the genetic changes that make Dahl SS rats a good model for studies of hypertension^38^ result in enhanced TRPC6 signaling somewhere within the animal (possibly but not necessarily within glomeruli). In this regard, Dahl SS rats typically exhibit slightly increased urine albumin excretion, even on low‐salt diets.^46^

In contrast to the pattern observed in Akita mice,^34^ there was no point in the course of our analyses where a protective effect of TRPC6 inactivation on urine albumin excretion or BUN could be discerned. Thus, urine albumin excretion was elevated at all of the time points following STZ treatment that we measured, (4, 8, and 10 weeks) and urine albumin excretion was indistinguishable in *Trpc6* ^wt/wt^ or *Trpc6* ^del/del^ rats. We cannot exclude the possibility that *Trpc6* ^del/del^ rats are at least partially insulin‐resistant by the time we started our experiments, which would negate protective effects in diabetes to a greater extent than in other disease models we have examined.^25^, ^27^

In summary, in the present study we did not obtain any evidence, suggesting that TRPC6 channels are useful targets for pharmacotherapy of DN. While TRPC6 inhibitors remain plausible targets for some glomerular diseases, for example in certain forms of FSGS,^1^, ^25^, ^27^ the data from animal models of DN are much less encouraging. The most robust result has been obtained by simultaneous knockout of all known diacylglycerol‐responsive TRPC channels, as might be expected to occur with a less selective channel inhibitor.^29^ However, the effect of combined inhibition of those channels on body weight essentially precludes consideration of that approach as a therapeutic strategy. The possibility that TRPC6 inhibition might promote insulin resistance^34^ is especially problematic in the context of DN. Finally, we note that while there are some similarities between the pathophysiology of DN and secondary FSGS, diabetes is a complex and systemic disease that simultaneously affects multiple cell types, including virtually every cell type in the kidney. This may also explain why we observed a protective effect of TRPC6 inactivation in chronic PAN nephrosis^25^ but failed to see any protection in STZ‐induced diabetes.

## CONFLICT OF INTEREST

The authors have nothing to disclose.

## AUTHOR CONTRIBUTIONS

N. Hassanzadeh Khayyat, E.Y. Kim, and S.E. Dryer designed the research. N. Hassanzadeh Khayyat performed the research. N. Hassanzadeh Khayyat analyzed data and prepared figures. N. Hassanzadeh Khayyat, S.E. Dryer, and E.Y. Kim wrote the paper.
