# Supplementary material for: TRPC6 inactivation does not protect against diabetic kidney disease in streptozotocin (STZ)‐treated Sprague‐Dawley rats
Source: FASEB Bioadv. 2019 Dec 2;1(12):773–82. doi: 10.1096/fba.2019-00077 (PMC6996301; doi:10.1096/fba.2019-00077)
Supplement: Supplementary file 1 [file FBA2-1-773-s001.pptx]

## Slide 1
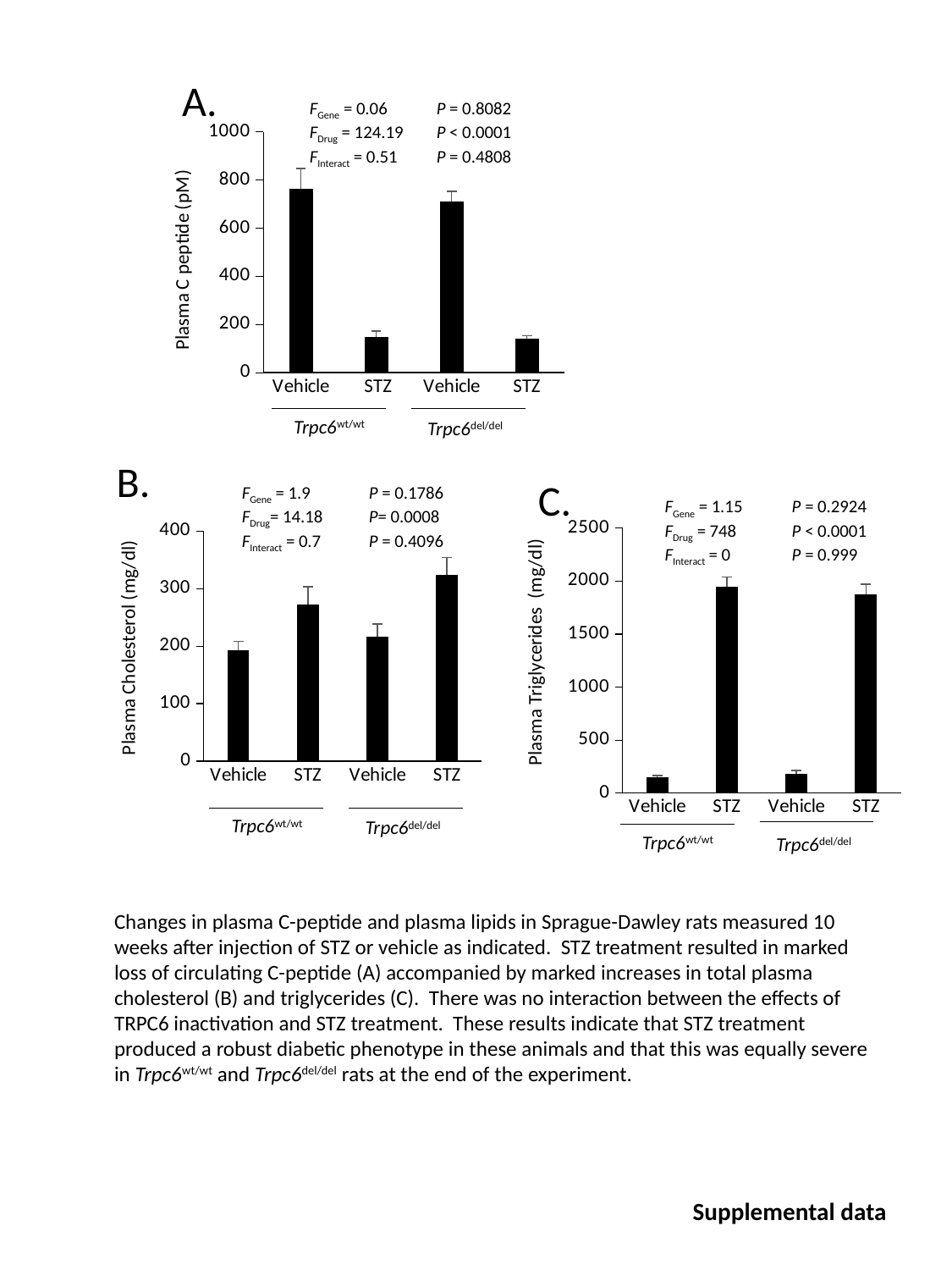

A.
FGene = 0.06 	P = 0.8082
FDrug = 124.19 	P < 0.0001
FInteract = 0.51 	P = 0.4808
### Chart
| Category | |
|---|---|
| Vehicle | 764.7574421188277 |
| STZ | 149.8682236466343 |
| Vehicle | 711.1071695370638 |
| STZ | 141.6622508527678 |Trpc6wt/wt
Trpc6del/del
B.
C.
FGene = 1.9	P = 0.1786
FDrug= 14.18 	P= 0.0008
FInteract = 0.7 	P = 0.4096
FGene = 1.15 	P = 0.2924
FDrug = 748 	P < 0.0001
FInteract = 0 	P = 0.999
### Chart
| Category | |
|---|---|
| Vehicle | 192.6604444444445 |
| STZ | 272.144875 |
| Vehicle | 216.4795555555554 |
| STZ | 324.2065714285689 |
### Chart
| Category | |
|---|---|
| Vehicle | 148.1134444444445 |
| STZ | 1943.5915 |
| Vehicle | 183.587888888889 |
| STZ | 1877.845428571429 |Trpc6wt/wt
Trpc6del/del
Trpc6wt/wt
Trpc6del/del
Changes in plasma C-peptide and plasma lipids in Sprague-Dawley rats measured 10 weeks after injection of STZ or vehicle as indicated. STZ treatment resulted in marked loss of circulating C-peptide (A) accompanied by marked increases in total plasma cholesterol (B) and triglycerides (C). There was no interaction between the effects of TRPC6 inactivation and STZ treatment. These results indicate that STZ treatment produced a robust diabetic phenotype in these animals and that this was equally severe in Trpc6wt/wt and Trpc6del/del rats at the end of the experiment.
Supplemental data
